# Reorganization Energy Predictions with Graph Neural Networks Informed by Low-Cost Conformers

**DOI:** 10.1021/acs.jpca.2c09030

**Published:** 2023-04-05

**Authors:** Cheng-Han Li, Daniel P. Tabor

**Affiliations:** † Department of Chemistry, Texas A&M University, College Station, Texas 77843, United States

## Abstract

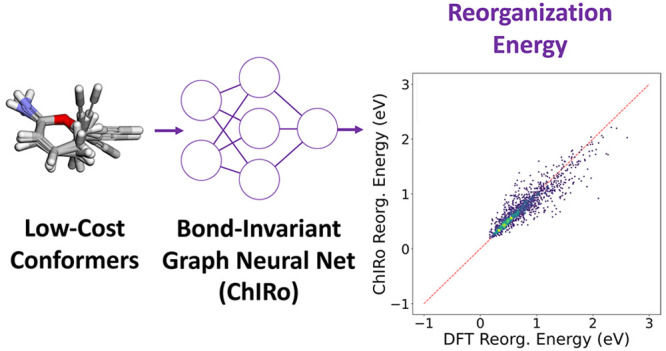

A critical bottleneck for the design of high-conductivity organic materials is finding molecules with low reorganization energy. To enable high-throughput virtual screening campaigns for many types of organic electronic materials, a fast reorganization energy prediction method compared to density functional theory is needed. However, the development of low-cost machine-learning-based models for calculating the reorganization energy has proven to be challenging. In this paper, we combine a 3D graph-based neural network (GNN) recently benchmarked for drug design applications, ChIRo, with low-cost conformational features for reorganization energy predictions. By comparing the performance of ChIRo to another 3D GNN, SchNet, we find evidence that the bond-invariant property of ChIRo enables the model to learn from low-cost conformational features more efficiently. Through an ablation study with a 2D GNN, we find that using low-cost conformational features on top of 2D features informs the model for making more accurate predictions. Our results demonstrate the feasibility of reorganization energy predictions on the benchmark QM9 data set without needing DFT-optimized geometries and demonstrate the types of features needed for robust models that work on diverse chemical spaces. Furthermore, we show that ChIRo informed with low-cost conformational features achieves comparable performance with the previously reported structure-based model on π-conjugated hydrocarbon molecules. We expect this class of methods can be applied to the high-throughput screening of high-conductivity organic electronics candidates.

## Introduction

Predicting the reorganization energy is crucial to accurately estimate the charge mobility of organic materials.^1−3^ The internal reorganization energy of the self-exchange electron transfer reaction (λ) often serves as a proxy in virtual screening campaigns that search for molecules promoting rapid electron transfer kinetics.^4−6^ Even one of the simplest reorganization energy approximations, Nelsen’s four-point method,^7^ requires both optimization and single-point calculations at neutral and charged states. These calculations are typically conducted with density functional theory. Since virtual screening campaigns often are conducted on thousands of molecules, an accurate and fast (compared to DFT or, ideally, semiempirical methods) reorganization energy predictor is needed to accelerate organic electronic materials discovery.

The increased throughput enabled by employing discriminative machine learning (ML) methods has found uses in many different workflows for molecular design and screening.^8−15^ Given its importance in organic electronics design, predicting the reorganization energy of molecular species has attracted increasing attention in the electronic molecule design community. Atahan-Evrenk et al.^16^ constructed a library of planar, fused-ring compounds and demonstrated the feasibility of making reorganization energy predictions on the library with deep neural networks trained on molecular descriptors. Abarbanel et al.^5^ trained a random forest model with hybrid descriptors for predicting λ on thiophene-based oligomers and then conducted a screening campaign for low-λ targets. Chen et al.^6^ investigated molecules up to 5 rotatable bonds with Δ-ML approaches for a virtual screening task where calculating λ depended on conformation.

Virtual screening campaigns often explore diverse chemical spaces, and thus, developing broadly applicable models (e.g., ones that take in universal features) would increase the throughput and utility of workflows that leverage virtual screening. The scope of chemical spaces in previous work is limited mostly to planar molecules and reorganization energies smaller than 2 eV.^4−6,16^ Developing models that predict the reorganization (or more generally, processes that involve the addition and removal of electrons) has been a challenging task, compared to tasks such as predicting the total electronic energy of the system. For the total electronic energy, deep neural networks informed by 3D structures of molecules have achieved state-of-the-art errors of  1 kcal/mol.^17−22^ For processes involving the addition and removal of electrons, AIMNET-NSE^17^ achieved an RMSE of ∼3.5 kcal/mol for predicting ionization potential and electron affinity on molecules up to 20 heavy atoms. This model incorporated molecular spin and charge into its architecture, and the training data involved samplings from molecular dynamics simulations to cover nonequilibrium structures.

Recently, graph neural networks (GNNs) have gained increasing interest in chemical applications.^18−22^ ChIRo,^22^ a 3D GNN that is invariant upon bond rotations, can encode different conformers in the same position in the latent space while still respecting the chirality of a molecule. The most salient difference between other 3D GNNs that have state-of-the-art accuracy on molecular benchmark sets (e.g., SchNet,^21^ DimeNet++,^18^ SphereNet^20^) and ChIRo is that the former employ multiple filters on bonds, angles, and torsions and layers of interaction blocks, which make the models sensitive to the molecular 3D geometry. This has been shown by the fact that it is more challenging for them to distinguish conformers in the contrastive learning task.^22^ For the purposes of this work, a given molecule in a functional-materials-oriented molecular screening campaign will be assigned single reorganization energy (the one associated with minimum energy geometries), and the most efficient screening procedures would be able to take any conformer of the molecule and predict the final reorganization energy that would be found with the four-point method (which requires geometry optimizations). In this case, for practical functional materials screening campaigns, being robust *against* variations in conformers is more important for the reorganization energy task.

In this work, we build a discriminative model based on the ChIRo architecture for predicting the reorganization energy of molecules from the QM9^23^ data set to determine if ChIRo is suitable for the reorganization energy prediction task, as QM9 contains diverse molecular scaffolds. We compare ChIRo with one of the relatively geometry-sensitive 3D GNNs mentioned above, SchNet, on the reorganization prediction task using structures from different levels of theory as input data. In addition, we compare its performance to a 2D GNN to gain insights into the importance of integrating three-dimensional information into a “conformer-robust” prediction task. Finally, we evaluate ChIRo on the π-conjugated hydrocarbon molecules to see how the model performs for the organic molecules with low reorganization energy in practice.

## Computational Methods

To generate the training set of geometries and energies, the SMILES strings of each molecule in the curated QM9 data set (the curation procedure described in Section S2 of the Supporting Information) were passed into Open Babel 3.1.1^24^ to generate initial geometries for searching conformers. We used CREST 2.1.1dev^25^ interfaced with xTB 6.3.3^26^ and the iMTD-GC^27^ workflow to identify at most ten conformational minima for each molecule. The same procedure was done for the neutral and cationic states of each molecule. Every conformer was further optimized at the B3LYP/def2-SV(P) level of theory using the Gaussian16 suite, version B.01,^28^ to find the lowest-energy geometries at neutral and cationic states. Once the lowest-energy geometries were found, λ can be estimated as^7^
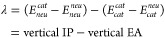
where *E* represents the total energy of the molecule under certain optimized geometry (subscript) and redox state (superscript), IP is ionization potential, and EA is electron affinity.

**Figure 1:**
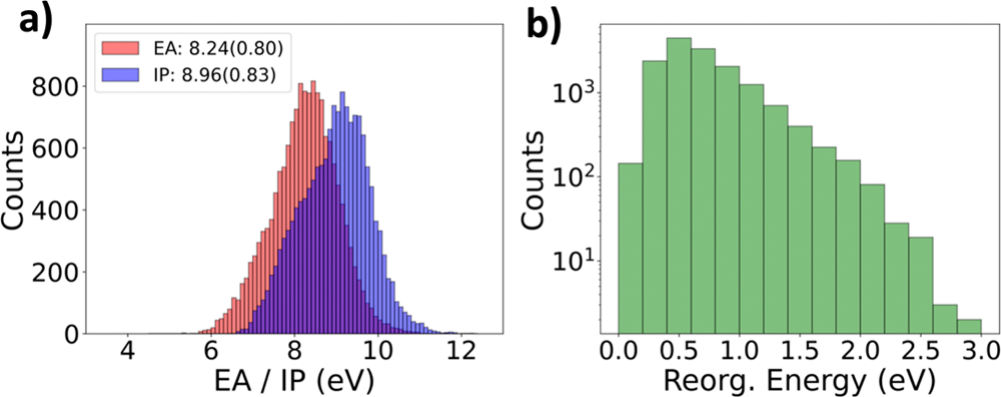
(a) Distribution of vertical electron affinity and ionization potential with their mean and standard deviation and (b) distribution of reorganization energy in the curated QM9 data set.

In total, the reorganization energies for 15,210 molecules were obtained. Distributions of vertical IP, vertical EA, and λ in this data set, where the maximum λ is about 2.94 eV, are shown in Figure 1. Among these molecules, we held out 10% of them as the fixed testing set, and the remaining molecules were split into training and validation sets with an 8:1 ratio repetitively. The full data set is available in our public repository at https://github.com/Tabor-Research-Group/fast_reorg_energy_prediction.

**Figure 2:**
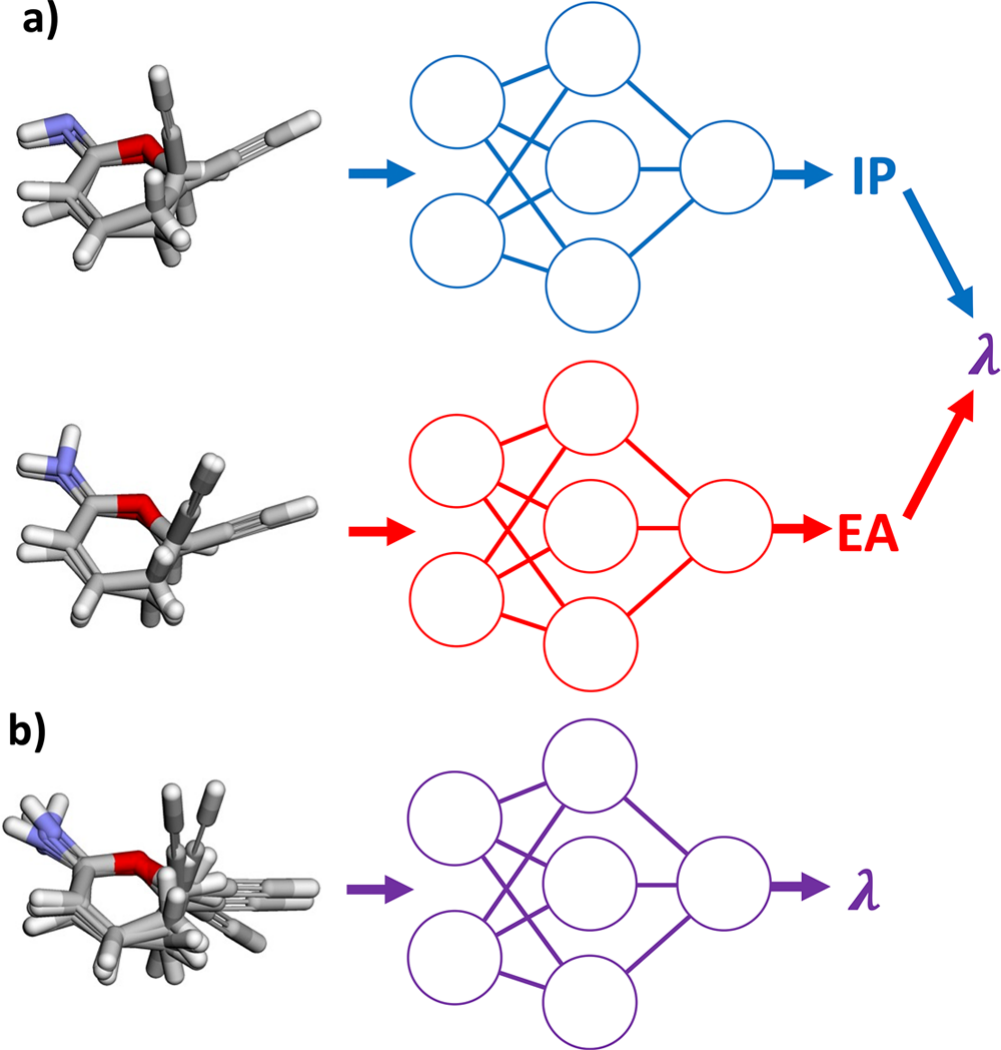
Scheme of predicting reorganization energy by (a) predicted vertical IP from neutral geometries minus predicted vertical EA from cationic geometries and (b) direct prediction of λ from RDKit geometries.

The relevant vertical IP and vertical EA for computing the reorganization energy were calculated from the neutral and cationic conformers, respectively (and independently). We trained two types of ML models for predicting vertical IP from neutral geometries and the vertical EA from cationic geometries and then combine the two results to obtain a predicted λ as shown in Figure 2a. Both ChIRo and SchNet were trained on either DFT geometries or CREST conformers to test their robustness on unseen molecules. Generating neutral and cationic semiempirical CREST conformers takes (on average) 2.7 CPU hours per molecule for the curated QM9 data set (substantially faster than DFT optimizations). Though improved from DFT, this timing is still computationally costly to get CREST conformers in a high-throughput virtual screening scenario, which usually involves thousands or millions of molecules.^14,29^

Aiming to further mitigate the geometry construction cost in the production stage of the λ predicting model, we randomly generated one RDKit^30^ conformer per molecule without further optimization in the *testing set* using the srETKDGv3 algorithm for reorganization energy predictions. The distance geometry-based conformer generation method^31^ implemented in RDKit is computationally efficient and incorporates empirical information (bond lengths, bond angles, torsional angles) to get molecular geometries. Embedding a molecule takes only 2.8 ms on average using a single CPU. Given these costs, our goal is to generalize reorganization energy predictions with low-cost RDKit-embedded geometries by leveraging the bond-invariant property of ChIRo. To compare the robustness of ChIRo and SchNet on RDKit-embedded geometries, we trained and tested both GNNs on the geometries at the same level of theory (DFT-optimized geometries or CREST conformers), then further tested the trained models on RDKit-embedded geometries (see Section S3 for the implementation details).

## Results and Discussion

Table 1 summarizes testing results for the reorganization energy prediction task between ChIRo and SchNet, where we repeated each entry five times across different training and validation splits (see Table S2 and Table S3 for testing results for predicting vertical IP and EA). Only the lowest-energy geometries at neutral and cationic states for each molecule were included when considering DFT-optimized geometries as inputs. SchNet outperforms ChIRo when both training and testing inputs are DFT geometries. However, if we assume that the lowest-energy λ is a molecular property and thus it is invariant to different conformations (as one would assume in certain virtual screening scenarios), the relative performance switches. In this case, the performance of ChIRo becomes superior when switching testing inputs to RDKit geometries. The testing errors of SchNet become higher than the standard deviation of the λ distribution. With this assumption, training GNNs to predict the same λ for different conformers from a molecule can be thought of as a regularization method.

**Table 1:** Performance of ChIRo vs SchNet for the Reorganization Energy Prediction Task Using Input Geometries at Different Levels of Theory

model	training inputs	testing inputs	MAE (eV)	RMSE (eV)	*R*^2^
ChIRo	DFT Geom.	DFT Geom.	0.130 (0.006)	0.183 (0.008)	0.763 (0.022)
	DFT Geom.	RDKit Geom.	0.218 (0.014)	0.340 (0.025)	0.174 (0.126)
	CREST Geom.	CREST Geom.	0.137 (0.003)	0.196 (0.005)	0.727 (0.014)
	CREST Geom.	RDKit Geom.	0.164 (0.005)	0.244 (0.013)	0.578 (0.045)
SchNet	DFT Geom.	DFT Geom.	0.093 (0.004)	0.128 (0.005)	0.883 (0.009)
	DFT Geom.	RDKit Geom.	0.391 (0.013)	0.536 (0.020)	–1.040 (0.150)
	CREST Geom.	CREST Geom.	0.132 (0.019)	0.183 (0.021)	0.759 (0.059)
	CREST Geom.	RDKit Geom.	0.336 (0.018)	0.564 (0.213)	–1.511 (2.094)

About two-thirds of molecules from the data set have more than one neutral or cationic CREST conformer (Distributions of the number of neutral and cationic CREST conformers for each molecule are shown in Figure S1 in Section S4). For the purposes of this implementation, the target vertical IP or EA should remain the same across different conformers when training GNNs. Although we can see higher testing errors for both ChIRo and SchNet for CREST conformers, they become more robust against RDKit geometries as expected. Here, SchNet does not generalize as well on RDKit geometries. In both cases, ChIRo performs better in predicting reorganization energy on low-cost RDKit geometries by introducing the invariance of bond rotations into its architecture.^22^ This design encodes the conformational flexibility of a molecule.

**Figure 3:**
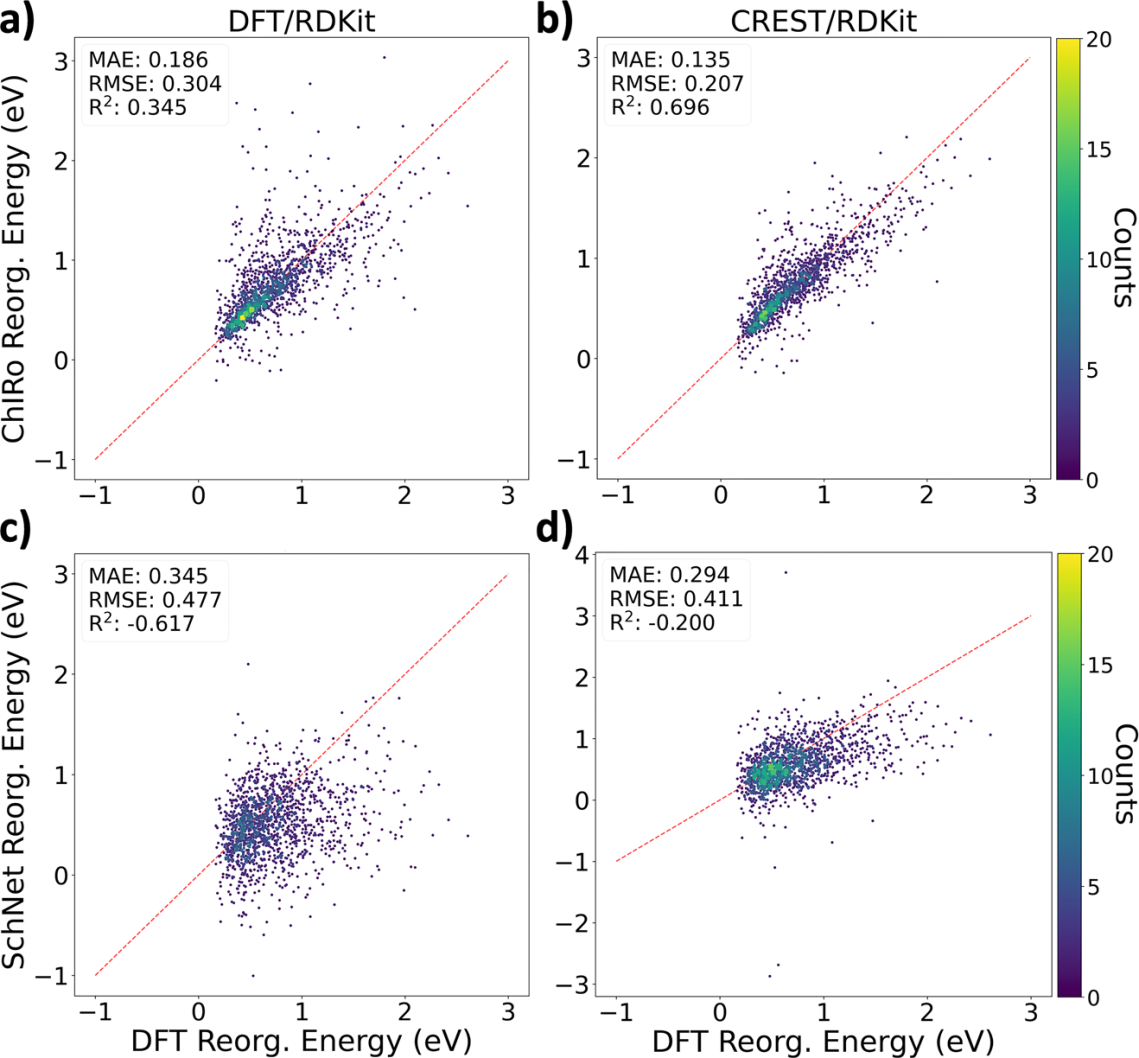
DFT reorganization energy versus predicted reorganization energy with RDKit geometries as inputs averaged from five ChIRo models trained on (a) DFT geometries and (b) CREST conformers and from five SchNet models trained on (c) DFT geometries and (d) CREST conformers.

In Figure 3, we show the predicted DFT reorganization energies in the testing set against outputs averaged across five GNNs with RDKit geometries as inputs (see Figure S2 for testing with DFT geometries (as a control) or CREST conformers as inputs). Averaging predictions from GNNs trained on different training/validation splits provides better performance on testing sets. While the points on the subplots converge toward the ideal fit line after switching training inputs from DFT geometries to CREST conformers for both ChIRo and SchNet, there are some outliers for SchNet trained on CREST conformers giving higher-than-usual and even negative reorganization energies (which is generally not seen). The negative predicted reorganization energies indicate some instances of poor generalization for RDKit geometries of GNNs trained on DFT geometries or CREST conformers.

We performed additional tests by generating five RDKit conformers per molecule for both ChIRo and SchNet to augment the data set and tested both models on the same RDKit geometries used above. Since there are no radical-cationic RDKit-embedded geometries, we trained GNNs to make direct predictions for λ as shown in Figure 2b. Here, a smaller starting learning rate was required (10^–4^) for training SchNet to prevent the gradient divergence, as the RDKit-embedding method can sometimes generate conformers far from the local minima of a molecule. Because SchNet does not separate bond-rotated geometries well, it is harder for SchNet to predict the same λ across very different conformers generated with low-cost methods. In contrast, ChIRo is suitable to be trained for predicting the same λ with different RDKit conformers as inputs. To verify this statement, we inspected the variance of the λ predictions from ChIRo and SchNet on the testing set with five RDKit conformers per molecule. It turns out that the average prediction variance for ChIRo (∼0.1 meV) is about 60 times smaller than SchNet (∼5.9 meV), which shows it is easier for ChIRo to predict similar λ from different conformers. We compared the performance between ChIRo and SchNet, as shown in Table 2 (see Section S5 for remaining testing results for ChIRo and SchNet). ChIRo not only outperforms SchNet but also gives better results than training and testing itself on DFT geometries, which is not the case for SchNet. Ensembles of ChIRo shown in Figure S3 also boost the performance on the reorganization energy prediction task.

**Table 2:** Performance of 2D and 3D GNNs for the Reorganization Energy Prediction Task Using RDKit Geometries as Inputs

Model	MAE (eV)	RMSE (eV)	*R*^2^
ChIRo	0.111 (0.002)	0.174 (0.003)	0.786 (0.008)
SchNet	0.152 (0.002)	0.214 (0.003)	0.674 (0.008)
MPNN	0.128 (0.004)	0.194 (0.004)	0.686 (0.022)

As an ablation study, we added a standard 2D GNN (MPNN^32^) implemented in an off-the-shelf package^33^ into the comparison. A 2D GNN does not take spatial information as input. In contrast, ChIRo, while being invariant to torsion changes, can still be variant to bond lengths and angles. In Table 2, the performance of MPNN is in between ChIRo and SchNet. This result indicates that even low-cost conformers can inform a GNN model for making λ predictions. In contrast to SchNet, ChIRo can be informed by the right mix of these low-cost 3D features because of its bond-invariant nature.

To go beyond the QM9 molecules and evaluate the applicability of ChIRo for organic electronic materials, we directly tested ChIRo trained with QM9 reorganization energy on the whole DFT reorganization energy data set provided by Chen et al.^6^ Then, we trained and tested ChIRo on this data set separately (see Section S6 for implementation details). There are 10,900 π-conjugated hydrocarbon molecules in this data set, where a series of molecular transformations were applied to benzene to create molecules of up to 60 atoms and five rotatable bonds. The range of the reorganization energy is 700 meV and the median is 269 meV. ChIRo trained with only QM9 reorganization energies initially gave a MAE of 334 meV on the π-conjugated hydrocarbon molecules, which is much higher than the standard deviation of the data set (ca. 80 meV). However, after retraining ChIRo on the π-conjugated hydrocarbon molecules, the performance of ChIRo using RDKit geometries as inputs reaches an accuracy (MAE = 40 meV) comparable to the accuracy of the purely structure-based model provided by the original study (MAE ∼ 37 meV).^6^ Although ChIRo did not generalize well on the π-conjugated hydrocarbon molecules initially, the performance can be improved by using molecules of interest as the training data. Thus, this type of model can be potentially used for organic electronic materials, which typically have lower reorganization energies.

## Conclusion

In this work, we built a reorganization energy data set from QM9 to cover a more diverse space of molecule moieties and a broader reorganization energy distribution for this study for predicting λ on low-cost RDKit geometries. With RDKit geometries as the testing inputs, we first compared the performance between ChIRo and SchNet trained on either DFT geometries or CREST conformers. Because of the sampling efficiency of conformers brought by the design of ChIRo’s architecture, ChIRo achieves better generalizability on low-cost RDKit geometries. While SchNet becomes worse on λ predictions when trained with RDKit geometries as inputs, ChIRo reaches lower testing errors than in the case of training and testing with DFT geometries. Comparison between 3D GNNs trained with RDKit geometries and a 2D GNN suggests the benefit of including molecular conformation as features and the importance of the symmetry of a model’s architecture. This shows the potential of training GNNs that cover conformational flexibility to make fast and accurate λ predictions for nonrigid molecules using geometries generated from low-cost methods. We further validated the viability of applying ChIRo with RDKit geometries on π-conjugated hydrocarbon molecules. This method can serve as one piece of a high-throughput molecular screening workflow for searching for candidates for organic electronics.

## Supporting Information Available

The Supporting Information contains: The Supporting Information is available free of charge at https://pubs.acs.org/doi/10.1021/acs.jpca.2c09030.

(1) Description of the contents of the raw data files on the GitHub repository, (2) curation procedure for the QM9 data set, (3) implementation details for ChIRo and SchNet, (4) distributions of the number of CREST conformers for neutral and cationic States, (5) testing results for vertical IP, vertical EA, and reorganization energy predictions, (6) implementation details for π-conjugated hydrocarbon molecule test, (7) π-conjugated hydrocarbon molecule test result, and (8) dispersion of reorganization energies in the curated QM9 data set (PDF)

## References

[ref1] Oberhofer H.; Reuter K.; Blumberger J. Charge Transport in Molecular Materials: An Assessment of Computational Methods. Chem. Rev. 2017, 117, 10319–10357. 10.1021/acs.chemrev.7b00086. 28644623

[ref2] Shuai Z.; Li W.; Ren J.; Jiang Y.; Geng H. Applying Marcus theory to describe the carrier transports in organic semiconductors: Limitations and beyond. J. Chem. Phys. 2020, 153, 080902 10.1063/5.0018312. 32872875

[ref3] Hsu C.-P. Reorganization energies and spectral densities for electron transfer problems in charge transport materials. Phys. Chem. Chem. Phys. 2020, 22, 21630–21641. 10.1039/D0CP02994G. 32969457

[ref4] Marques G.; Leswing K.; Robertson T.; Giesen D.; Halls M. D.; Goldberg A.; Marshall K.; Staker J.; Morisato T.; Maeshima H.; et al. De Novo Design of Molecules with Low Hole Reorganization Energy Based on a Quarter-Million Molecule DFT Screen. J. Phys. Chem. A 2021, 125, 7331–7343. 10.1021/acs.jpca.1c04587. 34342466

[ref5] Abarbanel O. D.; Hutchison G. R. Machine learning to accelerate screening for Marcus reorganization energies. J. Chem. Phys. 2021, 155, 054106 10.1063/5.0059682. 34364325

[ref6] Chen K.; Kunkel C.; Reuter K.; Margraf J. T. Reorganization energies of flexible organic molecules as a challenging target for machine learning enhanced virtual screening. Digi. Discovery 2022, 1, 147–157. 10.1039/D1DD00038A.

[ref7] Nelsen S. F.; Blackstock S. C.; Kim Y. Estimation of inner shell Marcus terms for amino nitrogen compounds by molecular orbital calculations. J. Am. Chem. Soc. 1987, 109, 677–682. 10.1021/ja00237a007.

[ref8] Janet J. P.; Kulik H. J. Machine Learning in Chemistry; American Chemical Society: Washington, DC, 2020.

[ref9] Butler K. T.; Davies D. W.; Cartwright H.; Isayev O.; Walsh A. Machine learning for molecular and materials science. Nature 2018, 559, 547–555. 10.1038/s41586-018-0337-2. 30046072

[ref10] Mater A. C.; Coote M. L. Deep Learning in Chemistry. J. Chem. Inf. Model. 2019, 59, 2545–2559. 10.1021/acs.jcim.9b00266. 31194543

[ref11] Dral P. O. Quantum Chemistry in the Age of Machine Learning. J. Phys. Chem. Lett. 2020, 11, 2336–2347. 10.1021/acs.jpclett.9b03664. 32125858

[ref12] Elton D. C.; Boukouvalas Z.; Fuge M. D.; Chung P. W. Deep learning for molecular design—a review of the state of the art. Mol. Syst. Des. Eng. 2019, 4, 828–849. 10.1039/C9ME00039A.

[ref13] Yang X.; Wang Y.; Byrne R.; Schneider G.; Yang S. Concepts of Artificial Intelligence for Computer-Assisted Drug Discovery. Chem. Rev. 2019, 119, 10520–10594. 10.1021/acs.chemrev.8b00728. 31294972

[ref14] Pollice R.; dos Passos Gomes G.; Aldeghi M.; Hickman R. J.; Krenn M.; Lavigne C.; Lindner-D’Addario M.; Nigam A.; Ser C. T.; Yao Z.; et al. Data-Driven Strategies for Accelerated Materials Design. Acc. Chem. Res. 2021, 54, 849–860. 10.1021/acs.accounts.0c00785. 33528245 PMC7893702

[ref15] Li C.-H.; Tabor D. P. Discovery of lead low-potential radical candidates for organic radical polymer batteries with machine-learning-assisted virtual screening. J. Mater. Chem. A 2022, 10, 8273–8282. 10.1039/D2TA00743F.

[ref16] Atahan-Evrenk S.; Atalay F. B. Prediction of Intramolecular Reorganization Energy Using Machine Learning. J. Phys. Chem. A 2019, 123, 7855–7863. 10.1021/acs.jpca.9b02733. 31204476

[ref17] Zubatyuk R.; Smith J. S.; Nebgen B. T.; Tretiak S.; Isayev O. Teaching a neural network to attach and detach electrons from molecules. Nat. Commun. 2021, 12, 4870 10.1038/s41467-021-24904-0. 34381051 PMC8357920

[ref18] Gasteiger J.; Giri S.; Margraf J. T.; Günnemann S. Fast and Uncertainty-Aware Directional Message Passing for Non-Equilibrium Molecules. arXiv; arXiv:2011.14115v3 [cs.LG]; 2022; https://arxiv.org/abs/2011.14115 (accessed 2023-02-06).

[ref19] Klicpera J.; Becker F.; Günnemann S. GemNet: Universal Directional Graph Neural Networks for Molecules; NeurIPS: 2021; https://openreview.net/forum?id=HS_sOaxS9K- (accessed 2023-02-06).

[ref20] Liu Y.; Wang L.; Liu M.; Lin Y.; Zhang X.; Oztekin B.; Ji S. Spherical Message Passing for 3D Molecular Graphs; ICLR: 2022; https://openreview.net/forum?id=givsRXsOt9r (accessed 2023-02-06).

[ref21] Schütt K. T.; Sauceda H. E.; Kindermans P.-J.; Tkatchenko A.; Müller K.-R. SchNet – A deep learning architecture for molecules and materials. J. Chem. Phys. 2018, 148, 241722 10.1063/1.5019779. 29960322

[ref22] Adams K.; Pattanaik L.; Coley C. W. Learning 3D Representations of Molecular Chirality with Invariance to Bond Rotations. ICLR. 2022; URL: https://openreview.net/forum?id=hm2tNDdgaFK (accessed 2023-02-06).

[ref23] Ramakrishnan R.; Dral P. O.; Rupp M.; von Lilienfeld O. A. Quantum chemistry structures and properties of 134 kilo molecules. Sci. Data 2014, 1, 140022 10.1038/sdata.2014.22. 25977779 PMC4322582

[ref24] O’Boyle N. M.; Banck M.; James C. A.; Morley C.; Vandermeersch T.; Hutchison G. R. Open Babel: An open chemical toolbox. J. Cheminform. 2011, 3, 33 10.1186/1758-2946-3-33. 21982300 PMC3198950

[ref25] Pracht P.; Bohle F.; Grimme S. Automated exploration of the low-energy chemical space with fast quantum chemical methods. Phys. Chem. Chem. Phys. 2020, 22, 7169–7192. 10.1039/C9CP06869D. 32073075

[ref26] Bannwarth C.; Caldeweyher E.; Ehlert S.; Hansen A.; Pracht P.; Seibert J.; Spicher S.; Grimme S. Extended tight-binding quantum chemistry methods. WIREs Comput. Mol. Sci. 2021, 11, e1493 10.1002/wcms.1493.

[ref27] Grimme S. Exploration of Chemical Compound, Conformer, and Reaction Space with Meta-Dynamics Simulations Based on Tight-Binding Quantum Chemical Calculations. J. Chem. Theory Comput. 2019, 15, 2847–2862. 10.1021/acs.jctc.9b00143. 30943025

[ref28] Frisch M. J.; Trucks G. W.; Schlegel H. B.; Scuseria G. E.; Robb M. A.; Cheeseman J. R.; Scalmani G.; Barone V.; Petersson G. A.; Nakatsuji H.; Gaussian16, Revision B.01. 2016; Gaussian Inc.: Wallingford, CT.

[ref29] Pyzer-Knapp E. O.; Suh C.; Gómez-Bombarelli R.; Aguilera-Iparraguirre J.; Aspuru-Guzik A. What Is High-Throughput Virtual Screening? A Perspective from Organic Materials Discovery. Annu. Rev. Mater. Res. 2015, 45, 195–216. 10.1146/annurev-matsci-070214-020823.

[ref30] RDKit: Open-source cheminformatics. https://www.rdkit.org (accessed 2023-02-06).

[ref31] Riniker S.; Landrum G. A. Better Informed Distance Geometry: Using What We Know To Improve Conformation Generation. J. Chem. Inf. Model 2015, 55, 2562–2574. 10.1021/acs.jcim.5b00654. 26575315

[ref32] Gilmer J.; Schoenholz S. S.; Riley P. F.; Vinyals O.; Dahl G. E. Neural Message Passing for Quantum Chemistry. Proceedings of the 34th International Conference on Machine Learning: Aug 06-11, 2017; Precup D., Teh Y. W., Eds.;, PMLR: Sydney, Australia, 2017.

[ref33] Li M.; Zhou J.; Hu J.; Fan W.; Zhang Y.; Gu Y.; Karypis G. DGL-LifeSci: An Open-Source Toolkit for Deep Learning on Graphs in Life Science. ACS Omega 2021, 6, 27233–27238. 10.1021/acsomega.1c04017. 34693143 PMC8529678

