# Supplementary material for: Reorganization Energy Predictions with Graph Neural Networks Informed by Low-Cost Conformers
Source: J Phys Chem A. 2023 Apr 5;127(15):3484–9. doi: 10.1021/acs.jpca.2c09030 (PMC10848248; doi:10.1021/acs.jpca.2c09030)
Supplement: Supplementary file 1 — jp2c09030_si_001.pdf [file jp2c09030_si_001.pdf]

# Electronic Supplementary Information for "Reorganization Energy Predictions with Graph Neural Networks Informed by Low-Cost Conformers"

Cheng-Han Li[a] and Daniel P. Tabor[a*]

[a] *Department of Chemistry, Texas A&M University, College Station, TX 77842 USA*

E-mail: daniel_tabor@tamu.edu

# Contents

**S1 Description of Contents of Raw Data Files** **S3**

**S2 Curation of the QM9 Dataset** **S4**

**S3 Implementation Details for ChIRo and SchNet** **S5**

**S4 Distributions of the Number of CREST Conformers for Neutral and Cationic States** **S6**

**S5 Testing Results for Vertical IP, Vertical EA, and Reorganization Energy Predictions** **S7**

**S6 Implementation Details for $\pi$-conjugated Hydrocarbon Molecule Test** **S10**

**S7 $\pi$-conjugated Hydrocarbon Molecule Test Results** **S11**

**S8 Dispersion of Reorganization Energies in the Curated QM9 Dataset** **S12**

**References** **S13**

# S1 Description of Contents of Raw Data Files

All the data, including input geometries and target properties for the curated QM9 dataset and code for performing curation, the modified ChIRo model, and training and evaluation scripts for ChIRo and SchNet are provided at the following GitHub repository:
https://github.com/Tabor-Research-Group/fast_reorg_energy_prediction.

# S2 Curation of the QM9 Dataset

To build our QM9 training set, we applied the following criteria to remove highly unstable molecules while retaining a diverse set of molecules.

1. Remove "uncharacterized molecules" defined in the original QM9 paper. (3054 molecules removed)

2. Remove molecules that failed RDKit[S1] SMILES canonicalization from SMILES provided by the original QM9 paper after DFT optimization. (699 molecules removed)

3. Remove molecules that failed graph isomorphism check between SMILES from GDB17[S2] and SMILES provided by the original QM9 paper after DFT optimization. (2 molecules removed)

4. Remove molecules having atomization energy per bond smaller than 100 kcal/mol using atomic and molecular energies provided by the original QM9 paper. (104964 molecules removed)

5. Retain molecules with at least one ring. (17546 molecules retained)

6. Remove molecules that failed the RDKit embedding method using the srETKDGv3[S3] algorithm. (10 molecules removed)

7. Remove molecules having bond(s) broken or rearrangement reactions after reorganization energy calculations described in the main text. (2326 molecules removed)

# S3 Implementation Details for ChIRo and SchNet

We followed the default model architecture and naming stated in the original ChIRo[S4] paper with the hyperparameters in Table. S1. We adjusted the node and edge features to the QM9 molecules and the reorganization energy prediction task. Node features include one-hot encodings of atom type (H, C, N, O, F), formal charge (-1, 0, 1), degree (1, 2, 3, 4), hybridization state ($sp$, $sp^2$, $sp^3$, unspecified), and aromaticity. Bond features include one-hot encodings of bond type (single, double, triple, aromatic), whether the bond is considered conjugated, and whether the bond is in a ring. We used SchNetPack[S5] to implement SchNet[S6] in this study. We built SchNet with 5 interaction layers. In each layer, there are 30 atomwise features, and 30 convolution filters with pairwise distances expanded on 20 Gaussians and 5 Å cosine cutoff. For all the targets, the loss function is the mean squared error function. We trained ChIRo and SchNet to minimize the loss function using the Adam optimizer with a batch size of 128. The learning rate was scheduled to reduce by 0.8 with no improvement in validation loss for 5 epochs until it reach $10^{-6}$. The model with the lowest validation loss is saved as the best model after training.

Table S1: Hyperparameters for ChIRo

| | |
|---|---|
| Node Features Dimension | 21 |
| Edge Features Dimension | 7 |
| All MLP Hidden Activations | LeakyReLU |
| All MLP Output Activations | Identity |
| EConv MLP Hidden Layer Size | 64 |
| EConv MLP # Hidden Layers | 1 |
| $\mathbf{h}_0$, $\mathbf{h}_T$ Dimension | 32 |
| $\mathbf{h}_t = 1,...,T-1$ Dimension | 64 |
| # GAT Layers | 3 |
| # GAT Heads | 4 |
| $f_d$, $f_\phi$, $f_\alpha$, $f_c$ Hidden Layer Size | 128 |
| $f_d$, $f_\phi$, $f_\alpha$, $f_c$ # Hidden Layers | 2 |
| $f_\psi$, $f_{\text{out}}$ Hidden Layer Size | 256 |
| $f_\psi$, $f_{\text{out}}$ # Hidden Layers | 2 |
| $\gamma_{\text{aux}}$ | 0.001 |

# S4 Distributions of the Number of CREST Conformers for Neutral and Cationic States

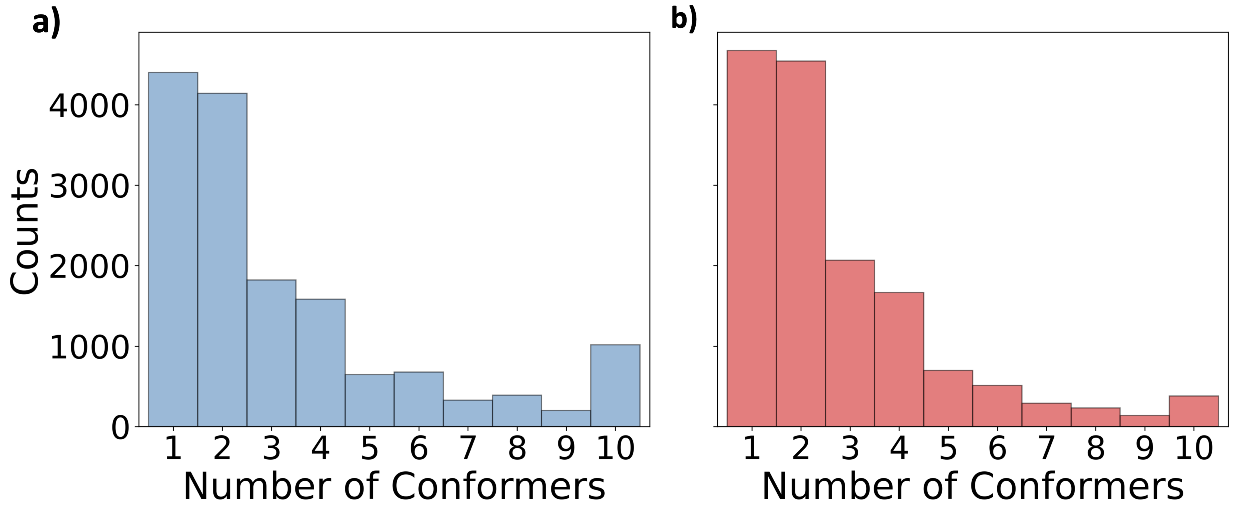


Fig. S1: Distribution of (a) neutral and (b) cationic CREST conformers for each molecule in the curated QM9 dataset.

# S5 Testing Results for Vertical IP, Vertical EA, and Reorganization Energy Predictions

Table S2: Prediction accuracy of ChIRo vs SchNet for vertical IP

| Model | Training Inputs | Testing Inputs | MAE (eV) | RMSE (eV) | $R^2$ |
|---|---|---|---|---|---|
| ChIRo | DFT Geom. | DFT Geom. | 0.084 (0.004) | 0.118 (0.007) | 0.979 (0.003) |
| | DFT Geom. | RDKit Geom. | 0.156 (0.016) | 0.284 (0.038) | 0.876 (0.036) |
| | CREST Geom. | CREST Geom. | 0.075 (0.001) | 0.108 (0.002) | 0.982 (0.001) |
| | CREST Geom. | RDKit Geom. | 0.105 (0.009) | 0.174 (0.025) | 0.953 (0.013) |
| | RDKit Geom. | RDKit Geom. | 0.066 (0.001) | 0.098 (0.003) | 0.985 (0.001) |
| SchNet | DFT Geom. | DFT Geom. | 0.068 (0.002) | 0.094 (0.002) | 0.987 (0.001) |
| | DFT Geom. | RDKit Geom. | 0.327 (0.010) | 0.452 (0.020) | 0.688 (0.028) |
| | CREST Geom. | CREST Geom. | 0.072 (0.002) | 0.102 (0.003) | 0.984 (0.001) |
| | CREST Geom. | RDKit Geom. | 0.308 (0.014) | 0.444 (0.039) | 0.699 (0.054) |
| | RDKit Geom. | RDKit Geom. | 0.112 (0.005) | 0.151 (0.005) | 0.965 (0.002) |

Table S3: Prediction accuracy of ChIRo vs SchNet for vertical EA

| Model | Training Inputs | Testing Inputs | MAE (eV) | RMSE (eV) | $R^2$ |
|---|---|---|---|---|---|
| ChIRo | DFT Geom. | DFT Geom. | 0.106 (0.008) | 0.152 (0.012) | 0.962 (0.006) |
| | DFT Geom. | RDKit Geom. | 0.169 (0.008) | 0.267 (0.011) | 0.884 (0.010) |
| | CREST Geom. | CREST Geom. | 0.117 (0.004) | 0.172 (0.006) | 0.952 (0.003) |
| | CREST Geom. | RDKit Geom. | 0.136 (0.011) | 0.209 (0.017) | 0.929 (0.012) |
| | RDKit Geom. | RDKit Geom. | 0.100 (0.002) | 0.153 (0.002) | 0.962 (0.001) |
| SchNet | DFT Geom. | DFT Geom. | 0.073 (0.005) | 0.104 (0.007) | 0.983 (0.002) |
| | DFT Geom. | RDKit Geom. | 0.301 (0.011) | 0.407 (0.015) | 0.732 (0.019) |
| | CREST Geom. | CREST Geom. | 0.116 (0.024) | 0.164 (0.027) | 0.956 (0.016) |
| | CREST Geom. | RDKit Geom. | 0.318 (0.016) | 0.525 (0.227) | 0.486 (0.494) |
| | RDKit Geom. | RDKit Geom. | 0.164 (0.005) | 0.224 (0.008) | 0.919 (0.006) |

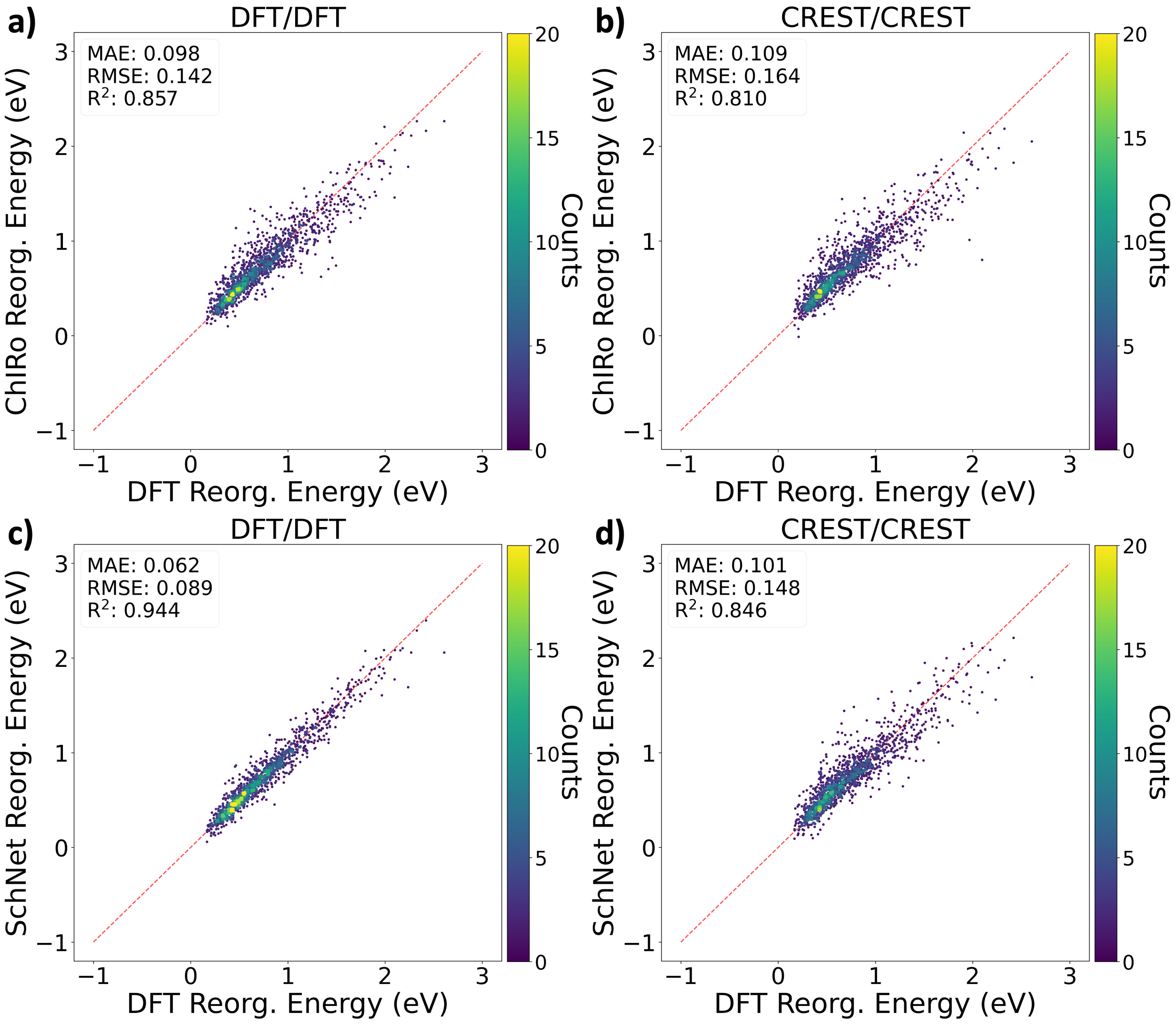


Fig. S2: DFT reorganization energy versus predicted reorganization energy averaged from five ChIRo models trained and tested on (a) DFT geometries and (b) CREST conformers, and five SchNet models trained and tested on (c) DFT geometries and (d) CREST conformers.

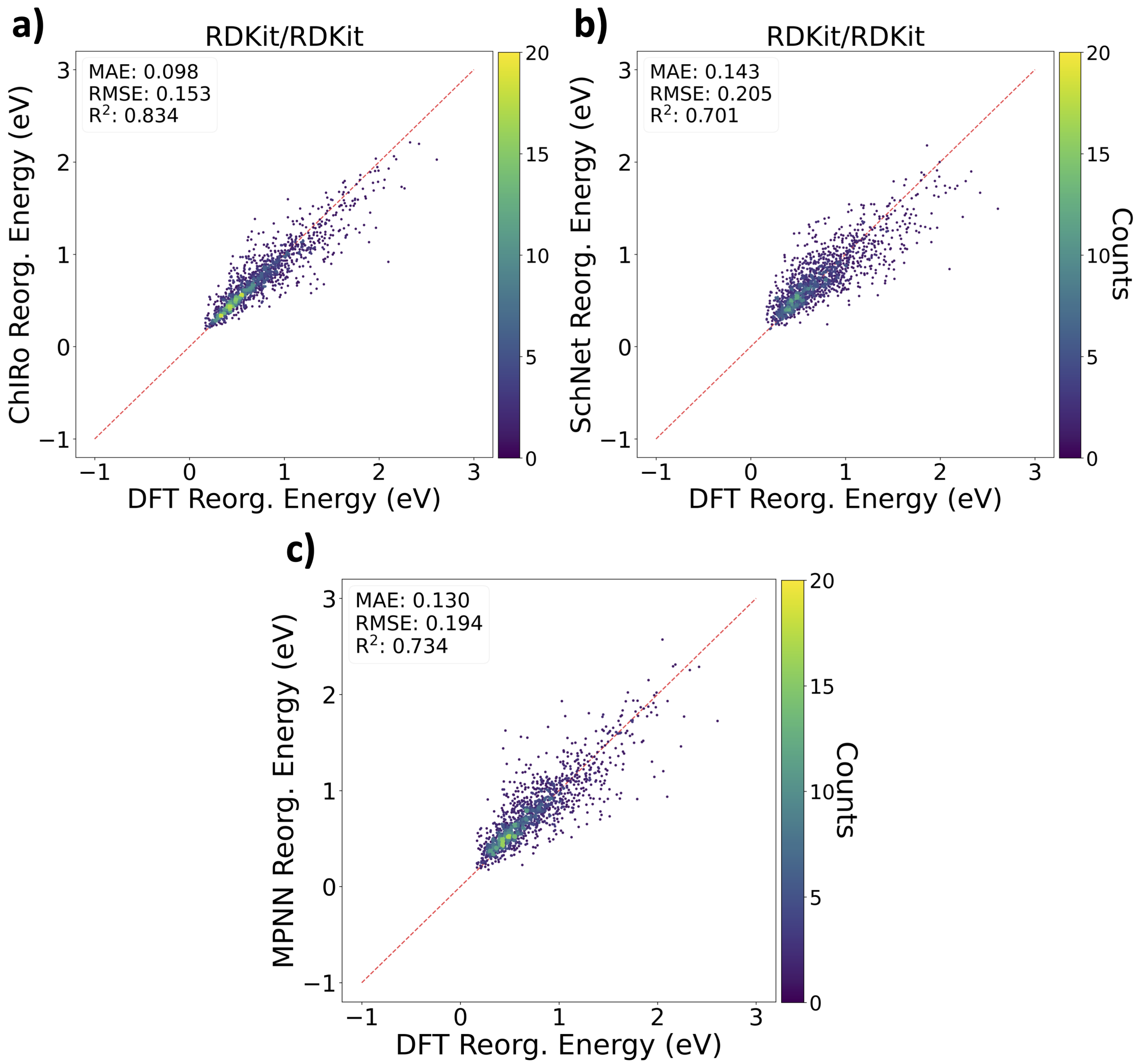


Fig. S3: DFT reorganization energy versus predicted reorganization energy averaged from five (a) ChIRo models and (b) SchNet models trained and tested on RDKit geometries, and (c) five MPNN models.

# S6 Implementation Details for $\pi$-conjugated Hydrocarbon Molecule Test

The initial dataset was downloaded from the GitLab repository[S7] provided by Chen *et al.*[S8] We used RDKit[S1] to read XYZ blocks provided in the dataset and determine the bonds and connections for each molecule. For testing ChIRo[S4] trained with QM9 reorganization energy, we took the average from the ChIRo ensemble as the predicted reorganization energy. To retrain ChIRo on the resulting $\pi$-conjugated hydrocarbon molecule dataset, the dataset was randomly split into training, validation, and testing sets with a 8:1:1 ratio. The training and validation sets were shuffled five times randomly to train a ChIRo ensemble, while the testing set was held fixed. Here, we used the same hyparameters and training protocol for the QM9 dataset, described in section S3, to train ChIRo on this dataset.

# S7 π-conjugated Hydrocarbon Molecule Test Results

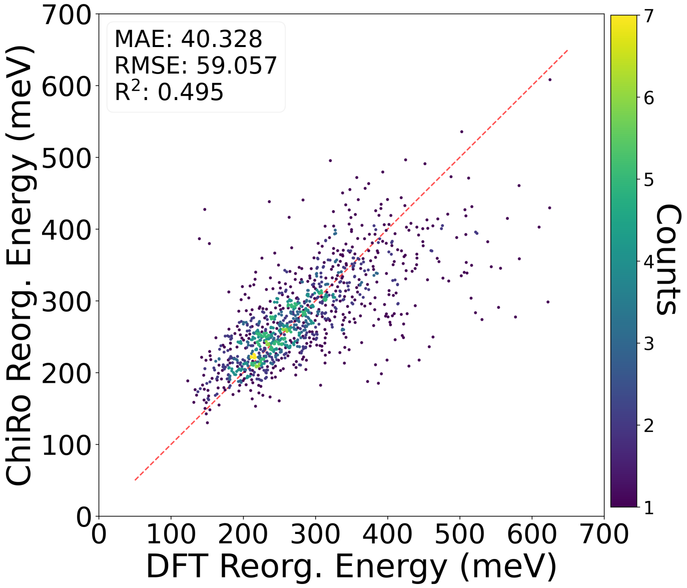


Fig. S4: DFT reorganization energy versus predicted reorganization energy averaged from five ChIRo models trained on π-conjugated hydrocarbon molecules.

## S8 Dispersion of Reorganization Energies in the Curated QM9 Dataset

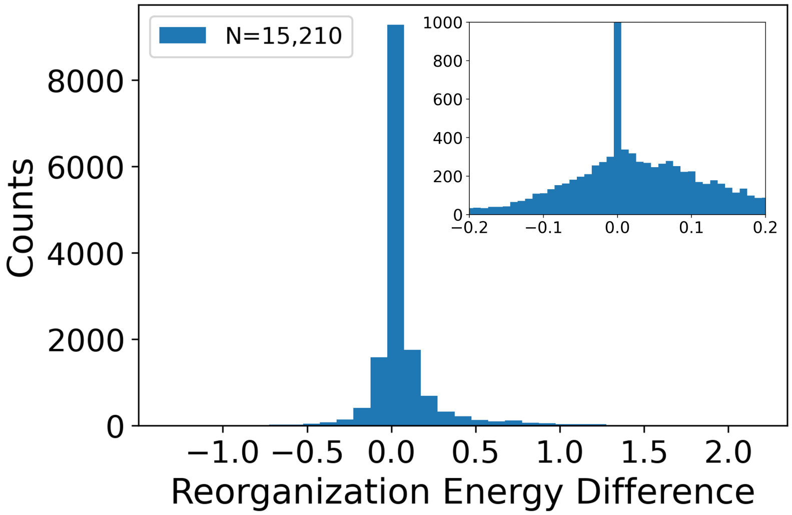


Fig. S5: Dispersion of reorganization energies in the curated QM9 dataset calculated by subtracting lowest-energy reorganization energy from the reorganization energy obtained from DFT-optimized cationic conformer with the highest energy. It should be noted that there are 4,591 molecules having zero difference due to the low number of conformers, as shown in Fig. S1.

# References

[S1] RDKit: Open-source cheminformatics. https://www.rdkit.org (accessed Feb 6, 2023).

[S2] Ruddigkeit, L.; van Deursen, R.; Blum, L. C.; Reymond, J.-L. Enumeration of 166 Billion Organic Small Molecules in the Chemical Universe Database GDB-17. *J. Chem. Inf. Model.* **2012**, *52*, 2864–2875.

[S3] Wang, S.; Witek, J.; Landrum, G. A.; Riniker, S. Improving Conformer Generation for Small Rings and Macrocycles Based on Distance Geometry and Experimental Torsional-Angle Preferences. *J. Chem. Inf. Model.* **2020**, *60*, 2044–2058.

[S4] Adams, K.; Pattanaik, L.; Coley, C. W. Learning 3D Representations of Molecular Chirality with Invariance to Bond Rotations. ICLR. 2022; URL: https://openreview.net/forum?id=hm2tNDdgaFK (accessed Feb 6, 2023).

[S5] Schütt, K. T.; Kessel, P.; Gastegger, M.; Nicoli, K. A.; Tkatchenko, A.; Müller, K.-R. SchNetPack: A Deep Learning Toolbox For Atomistic Systems. *J. Chem. Theory Comput.* **2019**, *15*, 448–455.

[S6] Schütt, K. T.; Sauceda, H. E.; Kindermans, P.-J.; Tkatchenko, A.; Müller, K.-R. SchNet – A deep learning architecture for molecules and materials. *J. Chem. Phys.* **2018**, *148*, 241722.

[S7] https://gitlab.mpcdf.mpg.de/kchen/oscs (accessed Feb 4, 2023).

[S8] Chen, K.; Kunkel, C.; Reuter, K.; Margraf, J. T. Reorganization energies of flexible organic molecules as a challenging target for machine learning enhanced virtual screening. *Digi. Discovery* **2022**, *1*, 147–157.
